# Extracorporeal Membrane Oxygenation in Acute Respiratory Failure due to Hemorrhagic Alveolitis in a Patient with Acute Myeloblastic Leukemia

**DOI:** 10.1155/2024/7571764

**Published:** 2024-03-18

**Authors:** Martina Cuccarelli, Lorenzo Schiavoni, Felice Eugenio Agrò, Giuseppe Pascarella, Fabio Costa, Rita Cataldo, Massimiliano Carassiti, Alessia Mattei

**Affiliations:** Department of Anesthesia and Intensive Care, Fondazione Policlinico Universitario Campus Bio-Medico, Rome, Italy

## Abstract

*Introduction*. Extracorporeal membrane oxygenation (ECMO) support for severe acute respiratory distress syndrome (ARDS) is nowadays widely used with notable results on the overall survival as reported in the ELSO registry near to 55% at 90 days. This is the reason why ECMO teams force the use of this extreme technique to several populations, even though there is still a lack of data about its use on hematological patients. *Case Report*. A 39-year-old woman without a history of previous diseases, but a new diagnosis of acute myeloblastic leukemia (AML) was admitted to intensive care unit (ICU) for worsening hypoxia and respiratory acidosis, presenting an ARDS with PaO_2_/FiO_2_ < 100 in spontaneous breathing treated with noninvasive ventilation via full-face mask. Meanwhile, chemotherapy was started leading to a severe bone marrow aplasia that was managed with multiple blood and platelet transfusions. These conditions did not allow physicians to start any invasive approaches. After 14 days, ARDS worsened whereas bone marrow recovered, making possible the beginning of an invasive mechanical ventilation, with low positive end-expiratory pressure and a low tidal volume. Moreover, an immediate extracorporeal CO_2_ removal (ECCO_2_R) therapy was added. Despite these efforts, no improvement was achieved, and that is why venovenous ECMO throughout femoral-jugular cannulation was applied. A full protective lung ventilation by ultralow tidal volumes was guaranteed. After 2 weeks of ECMO, a gradual weaning from ECMO support was started and completed after two days. No ECMO-related complications were registered. In the end, the patient started her weaning from the mechanical ventilation and reached 12 hours of spontaneous ventilation in oxygen therapy. *Discussion*. ECMO is used as a rescue therapy in patients affected by severe respiratory failure with life-threatening hypoxia and respiratory acidosis nonresponsive to other maneuvers. However, immunosuppression and coagulopathies of hematological malignancies are considered relative contraindications for ECMO, while long-lasting respiratory failure represents another relative contraindication to extracorporeal support. ECMO could be a valid option to improve the survival of hematological patients with severe ARDS and thrombocytopenia, and management could change case by case, even if high incidence of recurrency.

## 1. Introduction

Severe respiratory failure during the initial presentation of AML (acute myeloblastic leukemia) could be leukemia-related, hemorrhagic, or infective.

Respiratory support in immunocompromised patients must be balanced with high-risk mortality due to overinfection of respiratory tract promoted by respiratory devices, hindered respiratory clearance, and ineffective cough. Considering this, evidence suggests application of noninvasive ventilation in immunocompromised patients to avoid complications arising from endotracheal intubation and prolonged invasive ventilation [1].

Recent guidelines and evidence support a ultraprotective ventilatory management in ARDS patients, according to the strategy of respiratory rest. In this way, increasing invasive support in such fragile patients is both discouraged by large trials and supported by small experiences. However, none of the intensive care society has listed specific ventilatory management for immunocompromised patients, and the burden of choosing to continue treatment is entirely borne by the intensivist.

At the opposite, ECMO support for severe ARDS is nowadays widely used with notable results on the overall survival, as reported in the ELSO registry near to 55% at 90 days [2].

This is the reason why ECMO teams force the use of this extreme technique to several populations, such as elderly people, the obese, and oncohematologic patients [3].

Unfortunately, there is still a lack of data collected on these fragile patients, particularly on hematological ones, and a specific management of ARDS and extracorporeal support for each of these classes is not outlined, even though plenty of case reports and observational studies have been recently published reporting successful treatment despite comorbidities [4, 5].

The management of patients with AML on ECMO is really challenging because of immunosuppression, coagulopathy, and thrombocytopenia, which are traditionally considered as relative contraindications for ECMO, due to higher risk of bleeding and infections [6].

On the other hand, long-lasting respiratory failure with PaO_2_/FiO_2_ratio < 200, requiring assisted mechanical ventilation and subsequently invasive mechanical ventilation, throughout orotracheal intubation, for more than 7 days, represents another important contraindication to extracorporeal support.

## 2. Case Report

**Figure 1:**
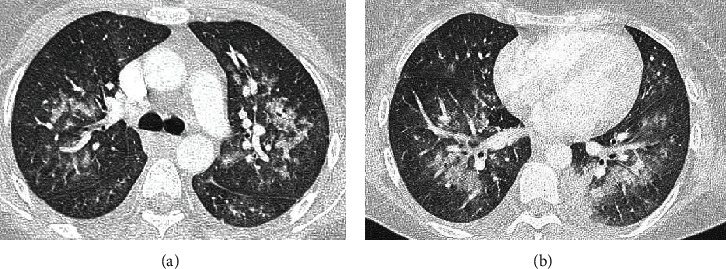
(a, b) CT images of hemorrhagic alveolar pneumonia at its onset at ICU admission.

A 39-year-old woman without a history of previous diseases was hospitalized for evidence of spontaneous ecchymoses and oral/nasal mucosal bleeding. Peripheral blood smear and bone marrow cytology revealed the presence of acute myeloblastic leukemia (AML), and she was admitted to intensive care unit (ICU) for worsening hypoxia and respiratory acidosis after an episode of chest pain and lung edema with hemoptysis, due to hemorrhagic alveolitis, as shown by chest computed tomography (CT) with bilateral floccular infiltrates like flowering tree (Figure 1).

However, the quick worsening of the patient's respiratory condition prevented from collecting alveolar samples so as to have a cytologic confirmation.

At her arrival to the ICU, the patient presented a severe anemia (Hb < 7 g/dl), neutropenia (WBC < 500 cells/mm^3^), and thrombocytopenia (platelets < 1000 cells/mm^3^) that were managed with multiple blood and platelet transfusions to keep a blood count of Hb > 7 g/dl, platelets > 3000, PT, and a PTT < 1.5, as hematologists recommended. This aplastic condition was the result of a first cycle of chemotherapy with cytarabine and daunorubicin started 4 days before and lasted for 21 days.

Moreover, the patient presented an ARDS with a PaO_2_/FiO_2_ ratio of minor then 100 in spontaneous breathing. At first, this respiratory failure was treated by applying a noninvasive mechanical ventilation throughout both the helmet and the full-face mask trying to hold a PaO_2_/FiO_2_ratio > 100.

Meanwhile, a prophylactic antibiotic therapy with piperacillin/tazobactam associated to linezolid was administered, as no cultures, both at the hematological medical department and even at ICU admission, were positive. At the same time, the administration of liposomal amphotericin B was started, but it was stopped beforehand in accordance with the hematologists for its high risk of collateral effects.

As a side effect of the severe thrombocytopenia, spontaneous vaginal, oral, nasal, and gastrointestinal bleeding episodes occurred and were treated with continuous infusion of tranexamic acid, norethisterone acetate, leuprolide acetate, vaginal packing, and platelet transfusion.

During this period, the patient developed some urinary tract infections by Candida species (albicans and parapsylosis) that were treated with caspofungin for 7 days.

Finally, WBC and platelets recovered, reducing the risk of both health care-related infections and uncontrolled bleeding due to invasive procedures.

However, after 14 days from ICU admission, respiratory conditions gradually worsened, and a severe ARDS (PaO_2_/FiO_2_ratio < 100) not responding to noninvasive mechanical ventilation occurred. This was the reason why an orotracheal intubation was performed, and then, an invasive mechanical ventilation was promptly started with low positive end-expiratory pressure (PEEP, 4 cmH_2_O) and low tidal volumes (TV, 320 ml) guided by diagnostic maneuvers evidencing very low opening pressure and early overstretching (near to 22 cmH_2_O) due to severe stiffness (static compliance near to 18 ml/cmH_2_O).

CT scans revealed a severe involvement of lungs with bilateral interstitial opacities mixed with bilateral consolidations.

However, after two days from orotracheal intubation, both lung stiffness (TV 270 ml, driving pressure 21 cmH_2_O, PEEP 3 cmH_2_O, respiratory rate 24 bpm, static compliance 12.8 ml/cmH_2_O) and the respiratory acidosis (PaCO_2_ of 79 mmHg) persisted determining a serious life-threatening condition, which led to the beginning of the ECCO_2_R therapy, with PrismaLung+© (Baxter, Deerfield, Illinois, USA) throughout a 14 Fr double lumen catheter (Two-Lumen Hemodialysis Catheter, CS-12142-F, Teleflex, Wayne, Pennsylvania, USA), while keeping a protective mechanical ventilation (TV 240 ml, respiratory rate 12 bpm, PEEP 4 cmH_2_O; FiO_2_ 0.6).

Despite five days on ECCO_2_R, no improvement was achieved on both oxygenation and hypercapnia, and for that critical condition, the venovenous ECMO support (Maquet RotaFlow©, Getinge, Rastatt, Germany) was applied by a femoral-jugular cannulation (HLS PVL 2555 and PAS 1915, Getinge, Rastatt, Germany).

**Figure 2:**
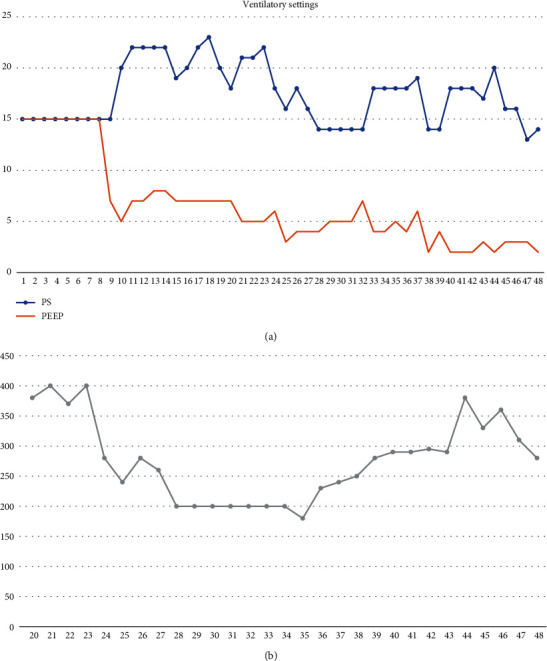
(a, b) Trends of ventilatory settings and ICU stay from admission to 47^th^ day. (a) On the 19^th^ day, the patient was intubated and managed with invasive mechanical ventilation. (b) TV has not been registered before day 19^th^, as the patient was supported with a noninvasive mechanical ventilation at first, and TV was not reliable under noninvasive mechanical ventilation.

Meanwhile, a full protective lung ventilation, through ultralow tidal volumes (TV 150 ml, near to 2 ml/kg), was guaranteed (Figure 2).

A prophylactic intravenous sodic heparin was administered at an infusion rate of 400 UI/h to avoid any thrombotic vascular events and even vv-ECMO circuit dysfunctions.

During the ECMO support, no platelet transfusion was needed as platelet count never fell to critical values. Such this event was interpreted as an indirect sign of bone marrow recovery.

Meanwhile, the patient unfortunately developed several infections, such as candidemia (C. parapsylosis) that was successfully treated with liposomal amphotericin B, and then, a ventilatory acquired pneumonia sustained by a Klebsiella pneumoniae carbapenemase-producing strain, treated with ceftazidime-avibactam.

**Figure 3:**
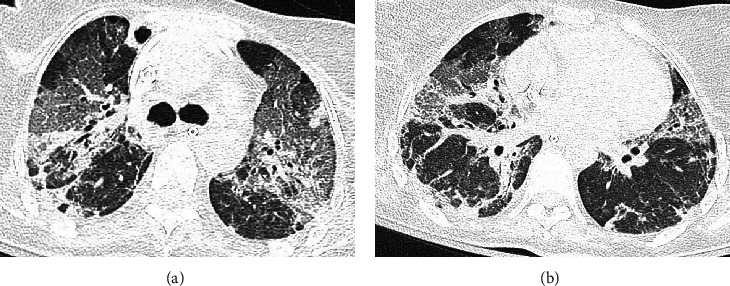
(a, b) CT images showing the switch to bilateral fibrosis of the lungs due to prolonged mechanical ventilation during the weaning trial from the venovenous ECMO.

On the 14^th^ day from the beginning of ECMO, as soon as both blood gas parameters and radiological imaging (Figure 3) revealed the ARDS complete resolution, a gradual weaning from ECMO was started and ended on the 16^th^ day of the ECMO maintenance. In the end, ECMO was removed after two days when the following parameters, PaCO2 38 mmHg, PaO2 101 mmHg, and PaO_2_/FiO_2_ 290, at the blood gas analysis were reached.

During the ECMO weaning trial, the patient achieved her autonomous breathing supported by mechanical ventilation in pressure support modality (PSV) with PEEP 3 cmH_2_O, PS 17 and a spontaneous TV over 300 ml with a static compliance near to 20 ml/cmH_2_O, and an adequate respiratory rate around 17 bpm. The ventilatory support for the weaning trial was managed without the guidance of an esophageal balloon, to avoid ventilatory-induced lung injury, and pressure support was derived from data acquired while the patient was deep sedated and curarized.

**Figure 4:**
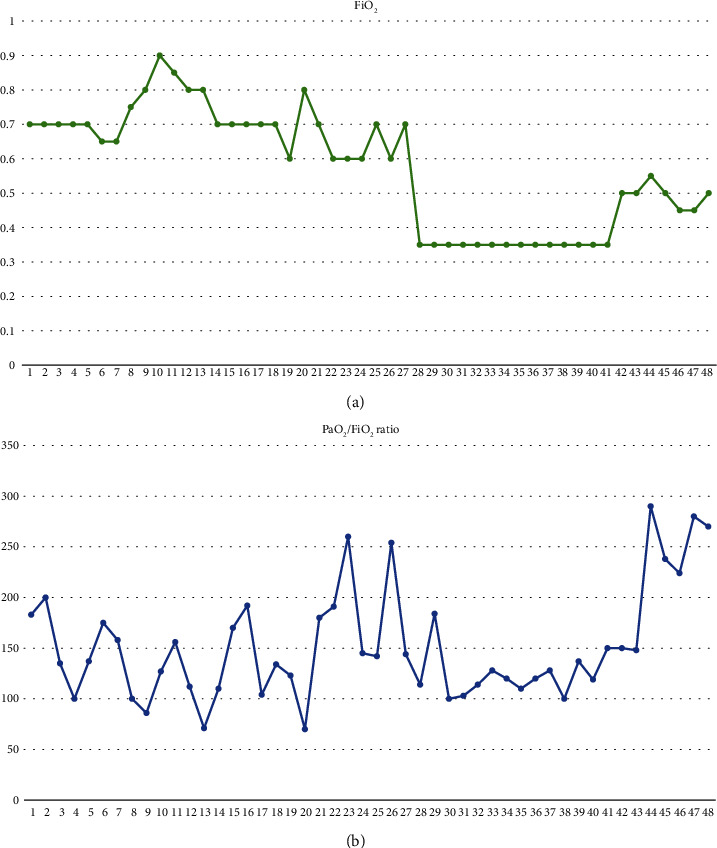
Trends of (b) PaO_2_/FiO_2_ ratio and (a) FiO_2_ during the first 48 days of ICU stay. Progressive decreasing of fraction of inspired oxygen matches with an increase in the PaO_2_/FiO_2_ ratio. Days of ECMO support are comprised between 28^th^ and 41^st^.

During the following weeks, the patient gradually underwent to an invasive mechanical ventilation weaning trial, obtaining more than 48 hours free from the airway support. Her improvement has been demonstrated by PaO_2_/FiO_2_ during recovery (Figure 4). However, she was never completely weaned from mechanical ventilation due to several episodes of ventilatory acquired pneumonia (VAP).

In fact, she developed a second episode of VAP caused by Klebsiella pneumoniae carbapenemase-producing strain, which was treated with meropenem-vaborbactam, but that overlapped, at the end of the patient's recovery, with Pseudomonas aeruginosa multidrug-resistant strain that required intravenous cefiderocol.

**Figure 5:**
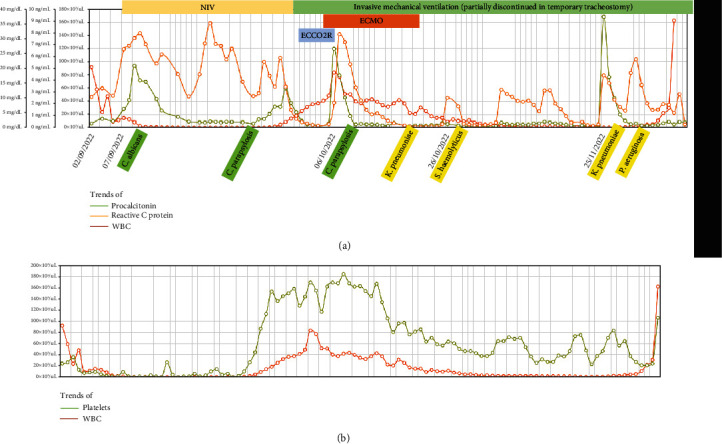
Trends of procalcitonin, reactive C protein, WBC, and platelets overlapped with a timeline of respiratory support, extracorporeal membrane oxygenation, and relevant nosocomial infections.

Major infections and trends of inflammatory markers have been scheduled in Figures 5(a) and 5(b).

For all this time, the patient underwent antifungal therapy with liposomal amphotericin B at full dosage to prevent any mycotic recurrences.

In the end, after 90 days from her ICU admission, she succumbed due to the recurrence of acute myeloid leukemia demonstrated by the last bone marrow biopsy with increased expression of nucleophosmin-1 and elevated hemopoietic precursor cellularity.

## 3. Discussion

Venovenous ECMO is commonly used as a rescue therapy in patients affected by severe respiratory failure with life-threatening hypoxia and respiratory acidosis, limiting lung injury due to high-pressure mechanical ventilation.

Severe respiratory failure during the initial presentation of AML could be leukemia-related, hemorrhagic, or infective [1].

However, the immunosuppression and coagulopathies typical of hematological malignancies are considered as relative contraindications for ECMO, due to a higher risk of bleeding and infections.

Moreover, our patient presented with no platelets and very low white blood cells on laboratory tests, due to complete response of bone marrow to chemotherapy.

On the other hand, long-lasting respiratory failure with PaO_2_/FiO_2_ratio < 200, requiring assisted mechanical ventilation and subsequently invasive mechanical ventilation for more than 7 days, represents another relative contraindication to extracorporeal support.

In this case, in consideration of severe hemorrhagic manifestations, we decided to avoid management of impaired coagulation on tracheal intubation and ECMO, starting a late extracorporeal support in a long-lasting mechanical ventilation. Literature and guidelines sustain that long-lasting ventilation patients are frequently exposed to lung fibrosis evolution that could worse ECMO weaning and recovery to spontaneous breathing, but high-volume ECMO centers have the potential to face these situations as revealed by recent numbers on ECMO support collected by the European Life Support Organization (ELSO) registry.

Our patient revealed very good adaptation to noninvasive ventilation for 14 days, obtained with moderate sedation with dexmedetomidine and remifentanil continuous infusion, with moderate PEEP (8 cmH_2_O) and low support pressure (16 cmH_2_O) with adequate ventilation and blood gas exchange, with moderate hypercapnia and mild hypoxia (PaCO_2_ 60 mmHg and PaO_2_ 90 mmHg).

As no infections were identified at admission, we deducted that ARDS could be related to hemorrhagic alveolitis, supported by radiological imaging, or caused by a hyperinflammation state due to AML.

Commonly, hemorrhage may also worsen during chemotherapy as a consequence of thrombocytopenia or disseminated intravascular coagulopathy [3]. Indeed, our patient showed several episodes of spontaneous bleeding while she was under chemotherapy, worsened either by APTT's stretch or the severe drop of the platelet amount, that we found in her daily blood tests. Those dramatic events made us in need of massive platelet transfusions (2 units per day) for 14 days.

Patients with underlying chronic immunosuppression or hematological malignancies treated with ECMO for ARDS have poor short- and long-term functional and survival outcomes with a mortality near to 50% and really affecting morbidity over the next year life. That is why ECMO support is usually contraindicated in these kinds of patients in many centers [3].

Real challenges in the management of patients with AML on ECMO are both coagulopathy and thrombocytopenia. Severe thrombocytopenia is seen in around 22% of patients requiring ECMO and is associated with more severe APACHE-II scores [3].

Current ELSO guidelines advise to maintain platelet count > 100 × 10^9^/L, but this threshold can be lower in adult patients, and often, this threshold cannot be reached or maintained [2].

Abrams et al. described transfusion triggers at lower levels < 20 × 10^9^/L if hemodynamically stable or <50 × 10^9^/L in patients with hemodynamic failure [7].

Yoon et al. described stopping heparin during ECMO in patients with platelet amount < 503 × 10^9^/L^6^, and local protocols follow these suggestions.

However, in literature, there are several cases of vv-ECMO and heparin maintenance despite of thrombocytopenia, and, since yet, none of the authors have reported a case of ECMO support in a patient with such a severe aplastic condition [8].

For example, there was a case of a 51-year-old smoker female affected by AML and ARDS linked to infective Klebsiella pneumoniae who underwent vv-ECMO with a platelet count < 39 × 10^9^/L [3].

In this case, we related our strategy to clinical manifestation of leukemia with spontaneous bleeding and waited for the acceptable recovery of bone marrow to start full intensive and invasive resuscitation.

A persistent thrombocytopenia with a total count under 1000 cells/mm^3^ despite of platelet transfusions was never reported in literature, and no suggestions could be obtained from guidelines and observational studies [9], but that severe condition led us to keep a conservative strategy until platelet recovery.

We switched to a much more intensive approach as soon as the platelet amount increased in a few days up to 140 × 10^3^/ml, and either a PTT or D-dimer count was stable (respectively, 25 s and 2 × 10^3^ ng/ml).

**Figure 6:**
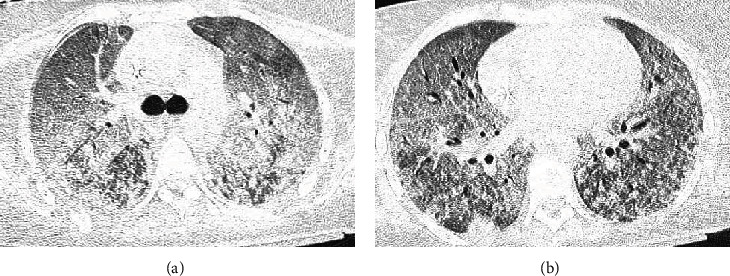
(a, b) CT images showing the dramatic worsening of the lung injury leading to the beginning of venovenous ECMO support to improve both oxygenation and CO_2_ removal.

Obviously, the patient paid this strategy developing lung fibrosis because of prolonged mechanical stress ventilation, and lung recovery was very slow but progressive (Figure 6).

Nowadays, there is still a lack of data related to the use of vv-ECMO in patients with AML receiving induction chemotherapy. In fact, in patients with a hematological malignancy, immunocompromised status and cytopenia, worsened by induction chemotherapy, could be further worsened by either ECMO cannulation or its maintenance, or other concurrent complications such as bleeding and bacterial, viral, and fungine infections.

However, usually after induction chemotherapy, acute lysis pneumopathy can lead to diffuse alveolar damage that can require ECMO support [6].

Current medical literature shows a case of a 44-year-old Asian male who was admitted to the ICU with the diagnosis of AML and started vv-ECMO for refractory hypoxemia while he was on induction chemotherapy [5].

This patient showed also a severe thrombocytopenia at the time of cannulation (platelets 4 × 10^9^/L) and that is why anticoagulation was not used during vv-ECMO [5].

Moreover, there is a case report about a 22-year-old man that was admitted to the ICU for ARDS linked to massive lysis pneumopathy during induction chemotherapy for AML [6].

ECMO could be a valid option to improve the survival of hematological patients with severe ARDS and thrombocytopenia both by reducing the lung injury related to high-pressure long-term ventilation and improving oxygenation. Thus, extracorporeal support needs to be evaluated without haste, appointing the origin of thrombocytopenia and its possible evolution to predict the risk of bleeding [8].

On the other hand, a late intervention could postpone chemotherapy administration and increase the rate of recurrency. Therefore, each case has to be managed for its specific clinical and hematological manifestations, and respiratory support has to be tailored to a single patient.

This clinical case suggests the reasonable efficacy of putting off ECMO in selected patients.

## Data Availability

All data were extracted from patient clinical report and are available from the first author upon request.
